# Editorial: Women in brain health and clinical neuroscience III: 2025

**DOI:** 10.3389/fnhum.2026.1827411

**Published:** 2026-03-27

**Authors:** Amanda Morato Do Canto, Sunaina Soni

**Affiliations:** 1 Faculty of Medical Sciences, State University of Campinas, Campinas, Brazil; 2 Departamento de Genetica Medica e Medicina Genomica, Universidade Estadual de Campinas, Campinas, Brazil; 3 Department of Physiology, AIIMS-CAPFIMS Centre, New Delhi, India

**Keywords:** brain, neuroscience, research, science, women

Ensuring diversity and representation in neuroscience is increasingly recognized as essential not only for equity but also for scientific excellence. Diverse research teams tend to ask broader questions, adopt more innovative approaches, and produce findings with greater clinical relevance. Although women now make up roughly half of graduate students in neuroscience, their representation declines markedly at higher academic ranks, comprising only about 25% of senior faculty and less than 15% of full professors in neurology and related fields. Despite progress at early career stages, disparities persist in authorship, funding, and leadership positions, limiting the full participation of women in shaping the direction of the field. The Research Topic “*Women in Brain Health and Clinical Neuroscience III*” was conceived in this context to showcase high-quality contributions led by women scientists and to encourage collaboration, mentorship, and greater visibility across clinical, cognitive, and translational neuroscience.

The four studies included in this Research Topic span complementary perspectives on brain function, ranging from non-invasive neuromodulation and neuropsychological characterization to large-scale brain network connectivity. Together, they emphasize the importance of understanding brain health at multiple levels—from cortical excitability and cognitive performance to symptom-related alterations in functional networks.

One contribution presents a detailed study protocol investigating whether high-frequency repetitive transcranial magnetic stimulation (rTMS) applied over the left dorsolateral prefrontal cortex modulates cortical excitability and enhances cognitive resilience in healthy older adults (Di Fazio et al.). By combining stimulation-derived motor-evoked potentials with comprehensive neuropsychological and emotional assessments, the protocol aims to characterize both physiological and behavioral effects of neuromodulation. A preliminary feasibility phase helped refine methodological procedures and evaluate tolerability, thereby laying the groundwork for a larger controlled trial. This preventive and mechanistic approach highlights the growing interest in using non-invasive brain stimulation not only as a therapeutic tool but also as a strategy to maintain and support cognitive function during aging.

Complementing this framework, a case report explores the feasibility of transcranial pulse stimulation (TPS) in a patient with treatment-resistant restless legs syndrome (Mitsi et al.). Following six sessions targeting cortical and subcortical network hubs, the patient reported substantial reductions in symptom severity, pain, and sleep disruption, with good tolerability. Although limited to a single case, this study introduces a novel network-oriented neuromodulation strategy and illustrates how innovative technologies may offer potential alternatives when conventional treatments prove insufficient. Such exploratory approaches are important first steps toward expanding the therapeutic toolbox for refractory neurological conditions.

From a neuropsychological perspective, another study examines executive functioning in patients with low-grade gliomas located in the supplementary motor area (Bala et al.). Using a comprehensive battery of cognitive tests, the authors identified deficits across several executive domains, including planning, inhibition, reasoning, switching, and cognitive flexibility. Performance was associated with tumor volume, lesion laterality, tumor subtype, and the presence of epileptic seizures, underscoring how focal structural pathology can disrupt distributed executive networks. These findings highlight the critical role of the Supplementary Motor Area in higher-order cognitive control and reinforce the importance of systematic cognitive assessment in neuro-oncology. By drawing attention to executive dysfunction that may otherwise remain underrecognized, the study emphasizes the need to integrate neuropsychological evaluation into routine clinical care for patients with low-grade glioma.

Extending the scope beyond primary neurological disorders, the final study investigates fatigue in individuals with Crohn's disease using resting-state functional connectivity analyses (McIver et al.). Compared with healthy controls, patients reported greater fatigue, which was associated with reduced functional connectivity between the superior parietal lobule and parahippocampal/hippocampal regions. Moreover, connectivity patterns moderated the relationship between disease activity and fatigue severity, suggesting that alterations in brain networks involved in sensorimotor integration and memory processing may contribute to symptom burden. By linking subjective experiences of fatigue to measurable neural correlates, this work expands our understanding of central nervous system involvement in systemic inflammatory disease and underscores the importance of brain–body interactions in shaping clinical outcomes.

Taken together, these contributions converge on several key themes: the central role of neural circuits in shaping cognition and behavior, the value of non-invasive technologies for both investigation and intervention, and the need to address functional outcomes that directly affect quality of life. They illustrate how clinical neuroscience benefits from integrating stimulation methods, cognitive assessment, and network-level imaging to better characterize brain resilience and dysfunction across diverse populations.

Beyond their scientific insights, the studies featured in this Research Topic also reflect the importance of initiatives that recognize and amplify the work of women researchers. Creating spaces that promote visibility, collaboration, and leadership contributes to a more inclusive scientific culture and ultimately strengthens the field as a whole. We would like to express our sincere appreciation to all authors who contributed their valuable research to this Research Topic, as well as to the reviewers whose thoughtful and constructive feedback helped maintain the high scientific standards of this Research Topic. We also acknowledge the support of the editorial and publishing teams who facilitated the successful completion of this issue. We hope that the studies presented here will stimulate further research, inspire new collaborations, and encourage greater participation of women scientists in brain health and clinical neuroscience. Ultimately, strengthening diversity and inclusion within the scientific community will enrich the field and accelerate progress toward a deeper understanding of the brain and improved care for individuals with neurological and psychiatric conditions.

