# Correction: Editorial: Women in brain health and clinical neuroscience III: 2025

**DOI:** 10.3389/fnhum.2026.1843983

**Published:** 2026-04-13

**Authors:** Amanda Morato Do Canto, Sunaina Soni

**Affiliations:** 1 Faculty of Medical Sciences, State University of Campinas, Campinas, Brazil; 2 Departamento de Genetica Medica e Medicina Genomica, Universidade Estadual de Campinas, Campinas, Brazil; 3 Department of Physiology, AIIMS-CAPFIMS Centre, New Delhi, India

**Keywords:** brain, neuroscience, research, science, women

Affiliation Department of Physiology, AIIMS-CAPFIMS Centre, New Delhi, India was omitted for author Sunaina Soni. This affiliation has now been added for author Sunaina Soni.

Author Sunaina Soni was erroneously assigned to affiliation Department of Medical Physiology, Swami Vivekanand Subharti University, New Delhi, India. This affiliation has now been removed for author Sunaina Soni.

The original version of this article has been updated.

